# MSmonitor-plus program and video calling care (MPVC) for multidisciplinary care and self-management in multiple sclerosis: study protocol of a single-center randomized, parallel-group, open label, non-inferiority trial

**DOI:** 10.1186/s12883-022-02948-z

**Published:** 2022-11-12

**Authors:** M. Hoving, P. J. Jongen, S. M. A. A. Evers, M. A. Edens, E. M. P. E. Zeinstra

**Affiliations:** 1 Multiple Sclerosis Center, Department of Neurology, Isala Hospital, Zwolle, the Netherlands; 2 Department of Health Services Research (HRS), Care and Public Health Research Institute (CAPHRI), Maastricht University, Maastricht, the Netherlands; 3 MS4 Research Institute, Nijmegen, the Netherlands; 4 Department of Health Sciences, University Medical Center Groningen and University of Groningen, Groningen, the Netherlands; 5 Trimbos Institute, Netherlands Institute of Mental Health and Addiction, Centre of Economic Evaluation & Machine Learning, Utrecht, the Netherlands; 6 Epidemiology Unit, Department of Innovation and Science, Isala Hospital, Zwolle, the Netherlands

**Keywords:** Multiple sclerosis, MSmonitor, Quality of life, MSmonitor-plus

## Abstract

**Background:**

We designed a new multi-modal version of the MSmonitor, called the MSmonitor-Plus and Video calling Care (MPVC), a self-management and education program with e-health interventions that combines frequent use of specific questionnaires with video calling in treating multiple sclerosis (MS) patients.

**Objective:**

To assess the effectiveness, cost-effectiveness and feasibility of MPVC compared to care as usual (CAU), with the goal of achieving equal or better quality of life for MS patients and their partners/informal caregivers.

Our hypothesis is that by using MPVC, monitoring will become more efficient, that patients’ self-efficacy, quality of life, and adherence to treatment will improve, and that they will be able to live their lives more autonomously.

**Methods:**

A randomized, parallel-group, open label, non-inferiority trial will be conducted to compare MPVC with CAU in MS patients and their partners/informal caregivers. A total of 208 patients will be included with follow-up measurements for 2 years (at baseline and every 3 months). One hundred four patients will be randomized to MPVC and 104 patients to CAU. Partners/informal caregivers of both groups will be asked to participate.

The study will consist of three parts: 1) a clinical effectiveness study, 2) an economic evaluation, and 3) a process evaluation. The primary outcome relates to equal or improved disease-specific physical and mental quality of life of the MS patients. Secondary outcomes relate to self-efficacy, efficiency, cost-effectiveness, autonomy, satisfaction with the care provided, and quality of life of partners/informal caregivers.

**Discussion:**

The idea behind using MPVC is that MS patients will gain more insight into the individual course of the disease and get a better grip on their symptoms. This knowledge should increase their autonomy, give patients more control of their condition and enable them to better and proactively interact with health care professionals.

As the consulting process becomes more efficient with the use of MPVC, MS-related problems could be detected earlier, enabling earlier multidisciplinary care, treatment or modification of the treatment. This could have a positive effect on the quality of life for both the MS patient and his/her partner/informal caregiver, reducing health and social costs.

**Trial registration:**

NCT05242731 Clinical Trials.gov. Date of registration: 16 February 2022 retrospectively registered.

## Introduction

### Background

Multiple Sclerosis (MS) is a chronic inflammatory, neurodegenerative disease of the central nervous system, which leads to a range of neurological deficits. It is most commonly diagnosed in young adult patients between the ages of 20 and 40. The symptoms often include fatigue, bladder, and visual disorders, pain, impaired mobility, spasticity, sexual restrictions, and psychological disorders such as anxiety and depression [1, 2]. MS patients should therefore be treated by multidisciplinary healthcare providers (HCP’s), including e.g. MS neurologists, MS nurse specialists, rehabilitation therapists, MS urologists, and neuropsychologists [3, 4].

Clinical disease monitoring of MS treatments has three elements: disease activity as manifested in relapses (reflecting inflammation), disability (reflecting neuro axonal loss), and functionality (reflecting the degree of compensation or cerebral reserve) [5]. To detect disease activity, it is recommended that MRI follow-up be started 3–6 months after treatment has been initiated, 6–12 months after the reference scan, and then annually. At each point, a standardized semi-quantitative comparator MRI should be used [5]. The disability and functionality are determined during the consultation with the MS neurologist or MS nurse specialist and can be managed by the patient himself/herself [6].

### Rationale

HCPs around the world are faced with the need to improve patient outcomes while controlling costs. Amongst other things, there is an increasing demand for chronic disease management for an aging population. Digital transformation, a process that aims to improve an entity by triggering significant changes to its properties through combinations of information, computing, communication, and connectivity technologies” [7], is critical for healthcare organizations to improve patient outcomes and reduce costs [8]. It could lead to improvements in diagnostics, prevention, and patient therapy, ultimately enabling HCPs to use a more evidence-based approach to improve clinical decisions as opposed to care as usual (CAU), where patients consult physically with their HCPs. Real-time interactions allow a doctor to monitor a patient ‘live’, rather than every few weeks or months [8]. Operational intelligence, integration-ready applications and platforms that help manage patients, networks and employees, are easily extensible and provide a consistent and intuitive user experience, enables efficient use of healthcare resources and services, optimizing costs with the goal of improving quality of life. Information technologies that enable this goal must be extensible, secure, reliable, affordable, and tailored to the individual organization’s level of digitization [9].

The therapeutic landscape for treating MS has been made more complex with the development of newer disease-modifying therapies (DMTs). These DMTs have given HCP’s and patients a wealth of options to better manage MS during the course of the disease [10]. A study from the United Kingdom (1998–2016) [11] has shown that treatment with early intensive therapy is beneficial. It is therefore important to start the right treatment on time [11].

New DMTs are more efficacious in reducing exacerbations and may even halt progression. However, side effects and risks (e.g., thyroid disorders) must be closely monitored. More consultations in the hospital for monitoring, are not recommended for MS patients because this will increase the fatigue that many MS patients experience. This fatigue in turn leads to worsening cognitive problems (a symptom of MS) such as forgetfulness and forgetting tasks. Decrease in cognition is unfavorable for consultation with the HCP.

Self-management, a future concept in healthcare, is a process by which patients take responsibility for changing their health-related behavior / healthcare by learning about their illness and managing the physical and psychosocial consequences [12]. Digital self-management applications are part of the digital transformation in healthcare [6, 12] and could lead to improvements in diagnostics, therapy, and prevention. In the case of complex, unpredictable, and chronically progressive diseases such as MS, it is being hoped that digitization and electronic health systems (e-Health) can help more effectively diagnose and monitor individual patients to enable optimal treatment [9, 13]. Most (especially younger) MS patients have a greater digital affinity and are quite proficient at indexing and using e-health services [14–16].

Several self-management applications for monitoring symptoms and signs already exist for MS patients, as well as diaries in which patients can keep private records of their symptoms and personal notes. Kidd ea. (2017), showed that self-management interventions can improve health-related quality of life in persons with MS, with some specific evidence of improvement in depression and anxiety symptoms [17]. There is also substantial evidence now that self-management interventions lead to health benefits in long-term conditions such as diabetes, arthritis, and heart disease [18].

### MSmonitor-plus and video calling care (MPVC)

We designed a new multi-modal version of MS monitor, called MS monitor-Plus and Video calling Care (MPVC), a self-management and /education program with e-health interventions that combines more frequent use of specific questionnaires with video calling in treatment and self-management of MS patients.

In this study, we will use the digital self-management application MSmonitor, which is NEN7510 and ISO27001 certified and developed in 2009 for MS patients with a focus on online self-measurements [19–21]. MSmonitor has an educational program with e-health intervention. MSmonitor provides insight into disease symptoms, helps achieve personal goals, and aims to increase the patient’s input in shared decision-making. Thus, the patient and the HCP can work efficiently and effectively together to obtain the best possible treatment.

MSmonitor provides patients and HCP’s with qualitative disease-specific and general patient-reported outcomes. The scores are automatically calculated and displayed on a dashboard. Changes are visible at a glance [22].

We have designed a new multi-modal version of MSmonitor, MSmonitor-Plus.

The consultations consist of MSmonitor-Plus appointments. MSmonitor-Plus appointments (completion of the specific questionnaires by the MS patients with interpretation and assessment by the HCP) are scheduled every 3 months.

Each nurse specialist has his/her own MS patients for whom they perform the checks. The results from the MSmonitor-Plus are in the Electronic Patient File.

In MPVC consulting and feedback can be given to the patient via the video calling program Beterdichtbij®. Beterdichtbij is a medical video calling program that complies with the guidelines of the Doctors’ Federation KNMG and is also NEN7510:2017 and ISO 27001 certified (Beterdichtbij, Utrecht, The Netherlands) [23]. Beterdichtbij program uses Microsoft Teams, a video calling program, which is integrated into the Electronic Patient File. In this process, the patient goes from the virtual waiting room to the consulting room, where the image connection starts with the HCP [24].

The MS Monitor-Plus and Beterdichtbij are integrated into the Electronic Patient Record for easy and convenient accessibility for the HCP. It is recommended that E-health applications are connected to the Electronic Patient File to ensure that text entries are efficient and do not increase the workload (retyping of text with risk of errors) [25, 26].

Through this development – the combination of an extended version of MSmonitor with the video calling program Beterdichtbij-, a new kind of MS care has been introduced. We will evaluate the (cost) effectiveness and feasibility of this combined digital approach to MS care.

The partner/informal caregiver of the MS patient will also have the opportunity to participate in this study. For some partners/ informal caregivers living with a MS patient is quite a task. Cognitive impairment and fatigue have a significant impact on patients. Therefore, we ask the partner/caregiver to complete specific questionnaires for partners/caregivers that are related to the partner/informal caregiver’s own life, such as finances, social contacts, work, health, and level of happiness. In addition, the partner/ informal caregiver completes the Cognition Failure Questionnaire (CFQ) about his/her partner with MS (observer-reported outcome). The MS patient completes the CFQ for him/herself (patient-reported outcome).

By participation of partners/informal caregivers, we hope to obtain more relevant information about MS disease, how a person is living his/her life with MS, and coping mechanisms.

The overall objective of this study is to assess the (cost) effectiveness and feasibility of MPVC compared to care as usual (CAU), with the goal of equal or better quality of life for MS patients and their partners/informal caregivers. Our hypothesis is that by using MPVC, monitoring will become more efficient (by completing MS-specific questionnaires in advance and by using video consultations), that patients’ self-efficacy, quality of life, and adherence to treatment will improve, and that they will be able to live their lives more autonomously.

## Methods

The study will consist of three parts: 1) a clinical effectiveness study, 2) an economic evaluation, and 3) a process evaluation.

### Research questions

Each part has its own research questions.

The operationalization is presented in more detail in Table 1: Constructs and validated questionnaires.

1. Clinical effectiveness

- Is MPVC, in comparison with CAU, equal or more effective in terms of quality of life (MSQOL-54), equal or more effective in terms of self-efficacy (MSSES) and other patient-reported outcomes, concerning adherence (adherence list MSmonitor), decrease in relapse frequency (Quick scan, questionnaire’ “Do I have a schub?) and severity (E-consulting, iMCQ), side effects of DMT (E-consulting, iMCQ), depression (HADS, Eq5d-5 L, iMCQ), anxiety (HADS, Eq5d-5 L), proactive coping (MSIS29, PIH, PTO) and autonomy (IPA)?
- Is MPVC, in comparison with CAU, equal or more effective in terms of improvement of the informal care situation, looking at burden of care (iVICQ), quality of life and happiness (CarerQol) for the informal caregiver?

2. Economic evaluation

- What are the cost-effectiveness and the cost-utility of MPVC in comparison with CAU from a societal perspective? (EQ-5D-5L, iPCQ)
- From a hospital perspective, is MPVC, in comparison with CAU, more effective in terms of quality of care (PTO, iMCQ) and lower consultation frequency with the HCPs?

3. Process evaluation

- Has MPVC been delivered in accordance with the protocol? And if not, what are the reasons for protocol deviation?
- What are the experiences and opinions of patients, informal caregivers and HCPs regarding MPVC?
- To what extent has MPVC impacted patients (i.e. do patients understand why it is relevant to work with MSmonitor-Plus), and has the MPVC impacted shared decision-making through consensual agreement between patient and doctor regarding the medical regimen?

**Table 1:** Constructs and validated questionnaires

**Construct**	**Operationalization**		
	**PROM by MS monitor Plus for patients**	**PROM abbreviation**	**Ref.**
MS-Bladder disorders	Screening bladder disorders (past 7 days)	Actionable	[27]
Quality of life of patients with urinary disorders in neurologic conditions	Short Form- Qualiveen	SF-Qualiveen	[28]
For individual tension strength	Checklist	Checklist	[29]
Functioning problems of MS and the perception of these problems. Proactive coping	Multiple Sclerosis Impact Profile	MSIP	[30]
Impact of MS. Proactive coping	Multiple Sclerosis Impact Scale (past 2 weeks)	MSIS-29	[31]
Bowel problems resulting from neurogenic disorders	Neurogenic Bowel Dysfunction Score	NBD	[32]
Decrease in relapse frequency. Proactive coping	The combination MFIS-5^a^, LMSQoL^a^, and Medication and Adherence Inventory. The combined use of MFIS-5, LMSQoL, and Medication and Adherence Inventory (“Quickscan”) enables quick, monthly assessments of health-related quality of life (HRQoL), fatigue experienced, medication, and DMD adherence	Quickscan	[19]
Anxiety and depression	Hospital anxiety and depression scale	HADS	[33]
Questionnaire on intimacy and sexuality in multiple sclerosis (MS) or spinal cord injury	The Multiple Sclerosis Intimacy and Sexuality Questionnaire	MSISQ-15	[34]
MS-specific quality of life	Multiple Sclerosis Quality of Life	MSQoL-54	[35]
Check PML and check schub	Natalizumab (Tysabri) checklist		[36]
Check schub	Questionnaire “Do I have a schub?”		[29, 37]
The patient can keep track of activities and moments of rest in order to gain insight into fatigue	Diary		[37]
**Construct**	**PROMs by RMp (eCRF), for patients**	**PROM abbreviation**	**Ref.**
Questionnaire for measuring subjective cognitive functioning. The items are globally related to memory and attention	The Cognitive Failure Questionnaire	CFQ	[38]
Patient’s perception of overall health	Euroqol Vertical Visual Analogue Scale	EQ VAS, versie 2.0	[39]
Health status: Mobility, Self-care, usual activities, pain/discomfort, anxiety and depression Economic appraisal of health	Euroqol-5 Dimensions with 5 Levels	Eq-5d-5L Versie 2.0	[40]
Severity of MS Health status Proactive coping	iMTA Medical Consumption Questionnaire	iMCQ	[41]
Effect of work on illness	iMTA Productivity Cost Questionnaire	iPCQ	[41]
MS-Participation and autonomy	Impact on participation and autonomy	IPA	[42]
Self-efficacy The MSSES-Function subscale measures confidence with functional abilities, whereas the MSSES-Control subscale measures confidence with managing symptoms and coping with the demands of illness	MS Self-Efficacy Scale	MSSES	[43]
Self-management of health Information on the disease, dealing with the consequences of chronic disease, active role in consultation, and the extent to which complaints and symptoms can be monitored at home	Partners in Health Scale (Working together on health)	PIH-NL	[44, 45]
**Construct**	**Prems by RMP (eCRF) for patients**	**PREM abbreviation**	**Ref.**
Satisfaction	Patient satisfaction questionnaire Isala	PTO	[46]
**Construct**	**Proms by consultation**	**PROM abbreviation**	**Ref.**
Severity of MS, Side effect DMT	E-consulting with neurologist/nurse specialist MS		

*PROM* Patient-reported outcome measure, *PREM* Patient-Reported Experience Measure, *Ref*. reference

^a^If applicable

### Study design

A single-center, randomized, parallel group, open label, non-inferiority study will be conducted to compare MPVC with CAU in MS patients (MonSter-1 study).

The intervention group will work with MSmonitor-Plus and Video calling Care (MPVC), the control group will receive CAU and maintain the number of outpatient consultations as applicable to the patient and will not use MPVC.

MS patients who do not participate in this study (e.g., because of a clear preference for the use of MVPC or for MSmonitor without video calling) will be asked to complete the baseline study questionnaires once. With this parallel study (MonSter-2 study), we can compare baseline characteristics in order to study the generalizability of the randomized controlled trial (RCT) outcomes.

**Fig. 1:**
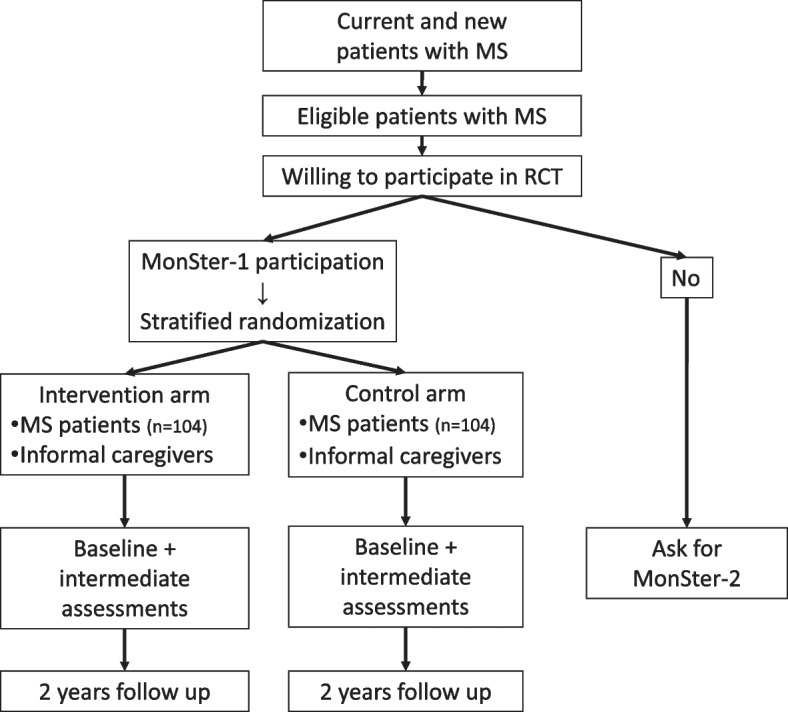
Flow chart of the study

Figure 1 presents the flow chart of the study. Tables 2 and 3 (assessment schedule) are schematic representations of the study design.

**Table 2:** Schedule of assessments concerning patients and caregivers in the intervention arm

PROMs			Standard time points									Variable time points
			B	3 m	6 m	9 m	12 m	15 m	18 m	21 m	24 m	
**ID**	**PROMs by MSmonitor Plus, for MS patients, with or without a caregiver/partner**											
1a 1b	Actionable SF-Qualiveen	Screening questionnaire for bladder disorders.	X	X	X	X	X	X	X	X	X	
		In order to assess health-related quality of life of patients with urinary disorders in neurologic conditions	X	X	X	X	X	X	X	X	X	
2	Checklist	For individual tension strength	X	X	X	X	X	X	X	X	X	
3	MSIP	Multiple sclerosis impact profile	X	X	X	X	X	X	X	X	X	
4	MSIS-29	Multiple sclerosis impact scale 29	X	X	X	X	X	X	X	X	X	
5	NBD	Neurogenic bowel dysfunction score	X	X	X	X	X	X	X	X	X	
6	Quick Scan	Five questions about the impact of fatigue on daily life, the Modified Fatigue Impact Scale-5 item Version (MFIS-5) and eight questions about health-related quality of life, the Leeds MS Quality of Life (LMSQol) and the registration of medication use	X	X	X	X	X	X	X	X	X	Every day if needed
7	HADS	Hospital anxiety and depression scale. Consists of a depression scale and anxiety scale, both are determined by means of seven questions	X		X		X		X		X	
8	MSISQ-15	Questionnaire on intimacy and sexuality in multiple sclerosis (MS) or spinal cord injury	X		X		X		X		X	
9	MSQOL-54	MS-specific quality of life	X		X		X		X		X	
10	Natalizumab checklist	16 questions every 28 days, submitted 2 days prior to the infusion of natalizumab	X									Every 28 days, if needed
11	Do I have a schub	Consists of 4 questions that the patient must answer. This shows whether he/she should contact the doctor to check if they have a schub	X									Daily available
12	Diary	The patient can keep track of activities and moments of rest in order to gain insight into fatigue	X									Daily available
ID	**PROMs by eCRF, for patients, with or without a caregiver/partner**											
13	iMCQ	iMTA Medical Consumption Questionnaire	X	X	X	X	X	X	X	X	X	
14	iPCQ	iMTA Productivity Cost Questionnaire	X	X	X	X	X	X	X	X	X	
15	CFQ	Cognitive failure questionnaire	X		X		X		X		X	
16	EQ-5D-5L	Health questionnaire (Dutch version)	X		X		X		X		X	
17	IPA	Impact on participation and autonomy	X		X		X		X		X	
18	MSSES	MS Self Efficacy Scale	X		X		X		X		X	
19	PIH	Partners in Heath Scale	X		X		X		X		X	
	**PREMs by eCRF for patients, with or without a caregiver/partner**											
20	PTO	Patient satisfaction questionnaire Isala	X				X				X	
	**PROMs by eCRF for caregivers/partners of MS patients**											
21	CarerQol-7D/VAS	Quality of life of informal caregivers for use in economic evaluations	X		X		X		X		X	
15	CFQ	Cognitive failure questionnaire	X		X		X		X		X	
22	iVICQ	iMTA Valuation of Informal Care Questionnaire	X				X				X	

Data collection was performed using MS monitor and eCRF. PROMs and PREMs are ordered by 1) frequency of administration and 2) alphabet

*B* baseline, *m* month

*PROM* Patient-reported outcome measure, *PREM* Patient-Reported Experience Measure

**Table 3:** Schedule of assessments concerning the control arm

ID	PROMs		Standard time points								
			B	3 m	6 m	9 m	12 m	15 m	18 m	21 m	24 m
	**PROMs by eCRF for MS patients, with or without a caregiver/partner**										
9	MSQOL-54	MS-specific quality of life	X		X		X		X		X
13	iMCQ	iMTA Medical Consumption Questionnaire	X	X	X	X	X	X	X	X	X
14	iPCQ	iMTA Productivity Cost Questionnaire	X	X	X	X	X	X	X	X	X
15	CFQ	Cognitive failure questionnaire	X		X		X		X		X
16	EQ-5D-5L	Health questionnaire (Dutch version)	X		X		X		X		X
17	IPA	Impact on participation and autonomy	X		X		X		X		X
18	MSSES	MS Self Efficacy Scale	X		X		X		X		X
19	PIH	Partners in Heath Scale	X		X		X		X		X
	**PREMs by eCRF for MS patients, with or without a caregiver/partner**										
20	PTO	Patient satisfaction questionnaire Isala	X				X				X
	**PROMs by eCRF for caregivers/partners of MS patients**										
22	iVICQ	Valuation of Informal Care Questionnaire	X				X				X
15	CFQ	Cognitive failure questionnaire	X		X		X		X		X
21	CarerQol	Quality of life	X		X		X		X		X

Data collection was performed using an eCRF

*B* baseline, *m* month

*PROM* Patient-reported outcome measure, *PREM* Patient-Reported Experience Measure

### Study population(s)

A total of 208 patients of all three types of MS, with or without their partner or informal caregiver will be included in the study (Tables 2 and 3). Most of the patients in the study have RRMS (relapses remitting, 80%), the least have PPMS (primary progressive, 8%) and the rest 10% SPMS (secondary progressive). This proportion corresponds to the prevalence of MS in the population of the Netherlands.

Inclusion criteria are 18 years of age or older, ability to understand Dutch and willingness and able to use MPVC.

Patients whose diagnosis of MS has not yet been clearly established or patients who have underlying diseases similar to MS in terms of symptoms (such as polyneuropathy, cerebral infarction, semi-lateral paralysis or other neurological diseases) will be excluded from participation.

The study team consists of the investigator, research nurse, the MS neurologist, and the MS nurse specialist.

### Setting

The study will be conducted at the MS Expertise Center of Isala Diaconessenhuis, Meppel location, Netherlands.

Isala is the largest general top clinical teaching hospital in the Netherlands with 6000 employees and 1100 beds. Isala is divided into 5 locations: Zwolle, Meppel, Steenwijk, Kampen and Heerde.

The study started in April 2021, followed by 1 year of inclusion and 2 years of follow-up. The last patient’s final study visit will be in 2024. The results should be finally analyzed in January 2025.

### Recruitment

Starting in April 2021, potential participants will be informed about the study during a consultation with one of the three MS neurologists, the nurse specialist or MS nurse. If patients are interested in participating in the study, their names will be placed on a trial list in our hospital’s Electronic Patient File (HIX). The coordinating investigator will contact the patient and explain the study. The coordinating investigator will ask if the participant has a partner/informal caregiver who would like to participate in the study. If the patient and eventual partner/informer caregiver agree, an Informed Consent Form (ICF) for the MS patient (ICF MS Patient) and – if applicable an ICF for the partner/informer caregiver (ICF partner/informer caregiver) - will be sent with a return envelope. The patient and partner/informal caregiver will be given 2 weeks to consider their decision to participate and if they have any questions, they can contact the investigator.

Upon receipt of the signed ICF, the coordinating investigator will sign the ICF and the patient and partner/informal caregiver will receive a copy. An appointment will be made for an inclusion session (by phone or face to face).

At baseline the patient and partner/informal caregiver will be registered in the electronic Case Report Form (eCRF) and will receive an email (the first) from the eCRF, immediately after enrollment with the initial invitation to fill out the research questionnaires.

Whether the research questionnaires were actually answered is tracked by the investigator. If research questionnaires are not completed on time, the patient and partner/informal caregiver will receive a reminder or are contacted by phone.

The intervention group will be signed up for the MSmonitor-Plus program and the nurse specialist will explain MPVC. A test appointment for a video calling connection via Beterdichtbij will also be made. Participants will receive an email from MSmonitor-Plus 4 times a year to complete questionnaires.

During standard MSmonitor-Plus appointments (every 3 months) the nurse specialist checks if the patient has completed the questionnaires. If the patient forgets to complete the questionnaires, the patient will be contacted by the nurse specialist (by phone or e-mail). The measurements and answers are recorded by the nurse specialist in the Electronic Patient Record.

### Sample size, concerning the effectiveness analysis

The sample size was based on detecting non-inferiority concerning the two subscales of the MSQol-54, i.e. the mental score and the physical score, at 2 years of follow-up.

We adopted the standard deviation (SD) of the baseline data of the Dutch MS Study [47] (largest SD, concerning preliminary data). The minimal clinically (non)important change (MIC) was estimate in accordance with a distribution-based approach.

Sample size calculation was performed using IBM SPSS SamplePower 3.0. Calculation with delta 8 points, SD 17.5, alpha 0.0125 (1-sided), and power 80% resulted in *n* = 93 required participants per arm. To compensate for 10% dropout, *n* = 104 participants per arm will be included in this study.

### Randomization

Stratified block randomization will be performed using the randomization module of our eCRF, ResearchManager (ResearchManager, Deventer, The Netherlands). ResearchManager is certified with ISO 27001 and NEN7510 and is affiliated with the 21 CFR part 11 FDA, which describes the administration of electronic records in a quality medical system of a medical device company.

Two strata will be used, i.e. sex and type of MS (RRMS, SPMS and PPMS). Distributions will be based on the known distributions in the Netherlands.

### Care as usual (CAU)

All patients in both study group receive care as usual. Care as usual consists first of making the correct diagnosis of MS (MRI scan, blood test and possibly lumbar puncture) and initiating the correct treatment method, adapted to the patient. MS care usually starts with DMT. Nearly all disease-modulating agents for MS are associated with suppression of the immune system that makes people with MS more susceptible to infections including viral infections. A tailor-made monitoring program (different per DMT) therefore applies to all these DMTs to control these risks as much as possible.

After starting a DMT, clinical and radiological monitoring should take place according to DMT-specific guidelines: In the first 2 years after the start of the treatment, an MRI will be made every 3–12 months. The check-ups in the hospital are 4 times a year after the starting the treatment: twice with the nurse specialist and twice with the neurologist. If the treatment is successful (no clinical or radiological disease activity), monitoring (in Isala) is performed twice a year by the neurologist and twice a year by the nurse specialist [4, 5].

The frequency of MRI monitoring is determined by the disease-modulating agent used. Furthermore, an MRI of the brain and spinal cord is required every 5 years. Blood tests are also frequently done, from once a month to every 6 months, depending on the type of DMT [4].

The MS patients come physically to the hospital and have a face-to-face consultation with their HCP.

### Intervention group: MPVC and CAU

The intervention group will use MPVC consisting of two E-health tools: 1) the MSmonitor-Plus and 2) the video calling program: Beterdichtbij [22, 23].

MSmonitor-Plus allows patients to complete questionnaires, related to MS and MS-specific quality of life. By completing these every 3 months, a schematic overview can be created of the complaints so that care and treatment can be adjusted.

Beterdichtbij is a specific, secure video calling program that is used in Isala. With Beterdichtbij physical consultations can be replaced by video calling.

Patients in the intervention group receive CAU and MPVC. For MPVC, patients complete specific questionnaires four times a year, in consultation with their HCP, and they engage in video calls with their HCP at least twice a year. The patient’s answers (responses) are graphically displayed over time and recorded in the Electronic Patient File by the HCP. These responses with observation points from the HCP (during consultation) and tests (such as an MRI) inform treatment and care.

### Control group: CAU without MPVC

In the control condition patients will have unrestricted access to CAU. Participants of the control group come to the hospital physically and have face-to-face consultations with the HCP’s, without using the MSmonitor-Plus and video calling. Medical support will be documented in the Electronic Patient File.

### Data collection methods

Concerning the intervention group, part of the data collection is embedded within MSmonitor-Plus. For the present study, additional research questionnaires are administered via the eCRF (Tables 2 and 3). Both groups, intervention and control group, fill in questionnaires in the eCRF, at baseline and every 3 months thereafter.

Participating partners/informal caregivers fill in specific validated questionnaires for partners/ informal caregivers in the eCRF at baseline and then every 6 months.

After randomization, a study number is automatically assigned to the participant (MS patient). The study numbers of the MS patients from the intervention group are also used in MSmonitor-Plus. These study numbers can be added manually in MSmonitor-Plus under the heading: ““Characteristics”“so that we keep the patient’s data together for final data collection.

The participants’ partner/informal caregiver will receive the same study number (filled manually) as his partner with MS, except for a letter P after the number (P=Partner) so that we can keep track of participants belonging together.

Parallel to the MS patient-reported data we collect in MSmonitor-Plus and in the eCRF, we will obtain objective data such as Expanded Disability Status Scale (EDSS) scores, MS relapse, Adverse Events (Side effects of the DMT, all unrelated to Multiple Sclerosis adverse events), hospitalizations (SAE) and all contact moments (MSmonitor-Plus consultations, number of physical appointments, video calls, telephone checks, check date of completed questionnaires). Every 3 months, the investigator will record these data for each MS patient in the eCRF and in the Electronic Patient File.

Confidentiality of all data is achieved through secure storage. The MS study team and the study monitor have access to the data. The static analysis will be based on a pseudo-analyzed dataset.

Ultimately, there are 18 different research (Table 4) patients: in the control group, with and without a partner/informal caregiver, patients in the intervention group, with and without a partner/informal caregiver and partners/caregivers of patients in the intervention group and control group with the corresponding type of MS.

**Table 4:** Overview of the research groups, with type of MS (RRMS, PPMS, SPMS)

**Intervention group (IG)**	**IG-RRMS**	**IG-PPMS**	**IG-SPMS**
MS patients (without partner/informal caregiver)	X	X	X
MS patients (with partner/informal caregiver)	X	X	X
Partners/informal caregivers of MS patients	X	X	X
**Control group (CG)**	**CG-RRMS**	**CG-PPMS**	**CG-SPMS**
MS patients (without a partner/informal caregiver)	X	X	X
MS patients (with a partner/informal caregiver)	X	X	X
Partners/informal caregivers of MS patients	X	X	X

*IG* intervention group, *RRMS* relapses remitting MS, *PPMS* primary progressive MS, *SPMS* secondary progressive MS, *CG* control group

Not every patient has a partner/informal caregiver or has a partner/informal caregiver who wants to participate in this study. Therefore, there will be more patients participating than partners/informal caregivers in this study.

### Outcomes

#### Clinical effectiveness

##### Primary outcome

1. Multiple Sclerosis Quality of Life (MSQOL-54). MSQOL-54 is a multidimensional measure of health-related quality of life that combines both generic and MS-specific items in a single instrument [48, 49]. This 54-item instrument generates 12 subscales along with two summary scores, and two additional single-item measures. The subscales are: physical functioning, role limitations-physical, role limitations-emotional, pain, emotional well-being, energy, health perceptions, social functioning, cognitive functioning, health distress, overall quality of life, and sexual functioning. The summary scores are physical health summary and mental health summary. The single item measures are satisfaction with sexual function and change in health.

The primary time point at which the effectiveness of MPVC is determined is 2 years.

#### Cost evaluation outcomes

Total costs over a 2-year period will be estimated using a bottom-up (or micro-costing) approach, where information on each element of service used is multiplied by an appropriate unit cost and summed to provide an overall total cost [50]. The time horizon will be the same as the follow-up period of the main study: from baseline and then every 3 months until a total period of 2 years.

We will assess intervention costs, healthcare costs, patient and family costs, and costs outside the health care sector. For this study, we will develop a cost questionnaire specially designed for this group, based on existing questionnaires [51] which will measure all relevant costs aspects.

To measure the actual use of resources, data will be obtained using combined sources (registrations by professionals and cost questionnaire). Resources used related to the interventions will be based on the registered time all professionals spent on the treatment. Intervention costs will include all the costs that will contribute to the development and administration of the MPVC and CAU, for example costs of training and travel expenses. All use of resources by the patient and their family in and outside the health care sector will be measured by means of a cost questionnaire (EQ-5D-5L), in which they continuously record volumes of resource utilization during the follow-up period.

Costs will be calculated by multiplying volumes (resource use) with unit costs. For units costs we will used the Dutch cost guidelines [6, 52, 53]. Productivity costs will be calculated by means of the friction cost method, based on a the mean added value of the Dutch working population. The friction costs method takes into account production losses confined to the period needed to replace a sick employee. In case of uncertainty, we will use a conservative estimation (i.e. the lowest cost price). Cost prices will be expressed in 2022 Euro. If necessary, existing cost prices will be updated to 2022 using the consumer price index. Resource use measurement (costs) will be measured continuously (for details see cost measurement), outcomes for the economic evaluation study will be measured at pre-test before randomization to the two study groups and every 3 months thereafter.

#### Process evaluation outcomes

The primary endpoint is the entire process of introducing MPCV over a 2-year period.

Process evaluation will be performed to assess whether MPVC was delivered in accordance with protocol, to examine the experiences and opinions of patients, caregivers and professionals regarding MPVC, and to determine to what extent MPVC has impacted adherence among patients. Process evaluation will be performed in accordance with the framework provided by Saunders et al [54]. This framework consists of a stepwise approach in which important characteristics for the process-evaluation plan are identified along seven basic components, i.e.: fidelity (quality), dose delivered (completeness), dose received (exposure), dose received (satisfaction), reach (participation rate), recruitment and context.

We will use a mixed method design in which both qualitative and quantitative data will be collected. The qualitative part will consist of observations during several group sessions over time. After each observation, a short interview will be held with the group leader(s) in which the group leader can reflect on his/her opinion regarding the session. During the last (sixth) group session of every MCI-group, a short evaluation form will be handed out to patients in which they will be asked to rate different aspects and themes of MPVC on a 7-point Likert scale. Furthermore, at the end of the study, focus groups will be held consisting of patients included in the study. The selection of participants will be based on maximal variation to get as many perspectives as possible. Participants will be selected based on age, gender, MS severity, cognition and effectiveness of the intervention (in order to compare patients in whom the intervention was successful versus unsuccessful). Focus groups will be conducted using a semi-structured questionnaire covering the topics identified in the framework provided by Saunders et al. [54].

### Statistical analyses

#### Clinical effectiveness analyses

Non-inferiority of MPVC compared to CAU, concerning both mental and physical scores of the MSQOL-54 at 2 years follow-up, will be analyzed using confidence intervals (1-tailed 98.75% or equivalently 2-tailed 97.5%).

When a confidence interval excludes the pre-defined non-inferiority margin of 8 points, non-inferiority will be concluded. In the case of substantial potential confounding (unbalanced baseline variables) however, analysis will be based on testing for non-inferiority to enable adjustment. If non-inferiority is demonstrated, superiority will be studied subsequently.

Courses of Proms between the two study arms will be compared using analyses for longitudinal data.

Analysis of primary outcomes will be performed in the per-protocol analysis set. Additional analyses will be performed in the (modified) intention-to-treat analysis set.

### Subgroup analyses

The effect of strata will be studied by stratified analyses.

### Missing data

Missing data will be managed by using modern techniques for the analyses of longitudinal data (Mixed Model analyses or Generalized Estimating Equations analyses).

### Cost effectiveness analyses

Economic evaluations compare additional costs and additional outcomes of CAU to MPVC. This economic evaluation will involve a combination of a cost-effectiveness analysis (CEA) and a cost-utility analysis (CUA). In a CEA effects are presented in clinical outcomes. The primary outcome measure for the cost-utility analysis will be QALYs, based on the Euroqol utility scores [55]. In the CUA, the Incremental cost-effectiveness ratio (ICER) will be expressed as the incremental costs per QALY. This economic evaluation will be performed from a societal perspective, which implies that all relevant costs and outcomes will be taken into account. The economic evaluation study will be performed in accordance with the Dutch Guidelines of the National Health Care Institute [56].

Following the Dutch guidelines, an annual discount rate of 1.5% will be applied to the effects, and future costs will be discounted to their present value by a rate of 4% [56]. Our primary (base-case analyses) will be performed in accordance with the intention-to-treat principle, including data from all participants regardless of whether they received the intervention or not.

A baseline analysis will be performed to examine the comparability of groups at baseline for both costs and outcomes. If necessary, methods will be applied to check for differences at baseline [57]. To investigate whether data are normally distributed, a Kolmogorov-Smirnov test will be performed. Despite the usual skewness in the distribution of costs, the arithmetic means will be generally considered the most appropriate measures to describe cost data [58, 59]. Therefore arithmetic means (and standard deviations) will be presented. In case of skewness of the cost data, non-parametric bootstrapping will be used to test for statistical differences in costs between the intervention and control group. Non-parametric bootstrapping is a method based on random sampling with replacement based on individual data of the participants. The bootstrap replications will be used to calculate 95% confidence intervals around the costs (95% CI), based on the 2.5th and 97.5th percentiles.

The incremental cost-effectiveness ratio (ICER) will be determined based on the incremental costs and effects of intervention and control groups. The cost-effectiveness ratio will be expressed in terms of cost per outcome gained, the cost-effectiveness ratio will focus on the net cost per QALY gained.

The ICER is calculated as follows. ICER = (Ci - Cc) / (Ei - Ec), where Ci is the annual total cost of both study groups, Cc is the annual total cost of the control group (treatment by usual means), Ei is the effect at three-year follow-up for the intervention and control group, and Ec is the effect at the last follow-up for the control group (treatment by usual means).

The robustness of the ICER will be checked by non-parametric bootstrapping. Bootstrap simulations will also be conducted in order to quantify the uncertainty around the ICER, yielding information about the joint distribution of cost and effect differences. The bootstrapped cost-effectiveness ratios will be subsequently plotted in a cost-effectiveness plane, in which the vertical line reflects the difference in costs and the horizontal line reflects the difference in effectiveness. The choice of treatment depends on the maximum amount of money that society is prepared to pay for a gain in effectiveness, which is called the ceiling ratio. Therefore, the bootstrapped ICERs will also be depicted in a cost-effectiveness acceptability curve showing the probability that intervention and control groups are cost-effective using a range of ceiling ratios. In The Netherlands, a ceiling ratio between at € 20,000, € 50,000 and € 80,000 per QALY exists, depending on the burden of disease [60].

Additionally, to demonstrate the robustness of our base-case findings, a multi-way sensitivity analysis will be performed. In the sensitivity analysis uncertain factors of assumptions in the base case analysis will recalculated in order to assess whether the assumptions have influenced the incremental cost-effectiveness ratio (ICER), for example by varying cost-prices and volumes between minimum and maximum [61].

### Process evaluation analyses

Results from open-ended questions will be categorized and analyzed. Semi-quantitative data will be analyzed using descriptive statistics (i.e., frequencies, mean, and median).

## Results

The first inclusion was April 27, 2021. By May 31, 2022, a total of 208 MS patients and 122 partners/caregivers had been included in MonSter-1 and 34 MS patients were participating in MonSter-2.

An amendment was made to extend the inclusion period by 1 month (because informed consents would still be received). The amendment was approved.

After final data analysis, the results will be described in a scientific article(s). Authorship will follow the ICMJE guidelines.

## Discussion

In this study we will assess the (cost) effectiveness and feasibility of MPVC compared to CAU, with the goal of achieving equal or better quality of life for MS patients and their partners/informal caregivers.

We want to put the responsibility for the evaluation of disability and functionality not only on the HCP, but also on the patients with MS, by giving them a greater role in recording their disability. By involving them in their care, the patients are given greater responsibility. By completing the MSmonitor-Plus questionnaires every 3 months, this procedure will become routine. HCPs view the MS monitor-Plus outcomes (lists, graphs) and gain a better insight into the patient’s actual condition. As a result, MPVC will improve the effectiveness of the consultation process resulting in improved quality of life for the patient and partner/informal caregiver.

For the HCPs, the introduction of MPVC will mean a change in the way they provide care.

The HCP must monitor patient’s completion of the questionnaires (on time) so that correct medical treatment and care can be provided. The HCP should keep in touch with the MS patient on this. This could be an extension of his task, as compared to CAU. Patients could forget to fill out the questionnaires in MSmonitor-Plus due to cognitive impairment.

This new type of care contrasts with a face-to-face meeting in which the provider discusses medical and care aspects in an often too restrictive time frame. The Multiple Sclerosis Impact Scale (MSIP) also allows for prioritization of care questions; contact time can therefore be used more efficiently which improves care.

The time required to achieve a confidential care situation in MPVC is likely to be different for each patient. For instance, there will be patients who do not complete questionnaires on time or still find it difficult to make this change in care. A trusting relationship between patient and HCP could be motivating in this process.

The digital transformation in healthcare will definitely continue. Whether 2 years is enough to see the benefits of this form of digital care (MPVC care) remains to be seen. Therefore, an extension of the observation period by at least 2 years would be recommended.

The various expected effects on autonomy, self-efficacy, improved care, improved DMT, all contribute to improved quality of life. Accordingly, these effects were chosen as the measure of the primary outcome of the study.

## Data Availability

Data available on reasonable request from the authors.

## Abbreviations

CarerQol: The Care-related Quality of Life instrument
CAU: Care as usual
CFQ: The Cognitive Failure Questionnaire
CG: Control group
CUA: Cost utility analysis
DMT: Disease-modifying therapies
eCRF: Electronic case report form
Eq5D-5L: Euroqol-5 Dimensions with 5 Levels
HADS: Hospital anxiety and depression scale
HCP: Health care provider
HIX: Healthcare Information eXchange
ICF: Informed consent form
ICER: Incremental cost-effectiveness ratio
IG: Intervention group
iMCQ: iMTA Medical Consumption Questionnaire
IPA: Impact on participation and autonomy
iPCQ: iMTA Productivity Cost Questionnaire
ISO: Internationale organisatie voor standaardisatie
iVCQ: iMTA Valuation of Informal Care Questionnaire
KNMG: Koninklijke Nederlandsche Maatschappij tot bevordering der Geneeskunst
METC: Medical Research Ethics Committee (Dutch: Medisch Ethische Toetsingscommissie)
MPVC: MSmonitor-Plus and Video Calling Care
MS: Multiple Sclerosis
MSQOL-54: Multiple Sclerosis Quality of Life
MSSES: MS Self-Efficacy Scale
MSIS29: Multiple Sclerosis Impact Scale
MSIP: Multiple Sclerosis Impact Profile
MSISQ-15: The Multiple Sclerosis Intimacy and Sexuality Questionnaire
NEN: NEderlandse Norm
NBD: Neurogenic Bowel Dysfunction Score
QALY: Quality-adjusted life year
RRMS: Relapsing Remitting Multiple Sclerosis
SPSS: Statistics is a powerful statistical software platform.
PPMS: Primary Progressive Multiple Sclerosis
PROM: Patient Reported Outcome Measures
PREM: Patient-Reported Experience Measure
PIH: Partners in Health Scale (Working together on health)
PTO: Patient satisfaction questionnaire Isala
SPMS: Secondary progressive multiple sclerosis
VAS: Visual Analogue Scale
